# Chemical Composition of Pyroligneous Acid Obtained from Eucalyptus GG100 Clone

**DOI:** 10.3390/molecules23020426

**Published:** 2018-02-15

**Authors:** Alexandre S. Pimenta, Maíra Fasciotti, Thays V. C. Monteiro, Kássio M. G. Lima

**Affiliations:** 1 Agricultural Sciences Academic Unit, Forest Sciences Graduate Program—PPGCFL, Forest, Bioenergy and Environment Research Group, Federal University of Rio Grande do Norte—UFRN, Natal 59280000, Brazil; aspimenta@ufrnet.br; 2 National Institute of Metrology, Quality and Technology, Duque de Caxias 25250020, Brazil; mfasciotti@inmetro.gov.br (M.F.); tvmonteiro-faperj@inmetro.gov.br (T.V.C.M.); 3 Institute of Chemistry, Chemistry Graduate Program, Biological Chemistry and Chemometrics Research Group, Federal University of Rio Grande do Norte—UFRN, Natal 59280000, Brazil

**Keywords:** pyroligneous acid, wood vinegar, Eucalyptus GG100 clone, GC-MS characterization

## Abstract

The present study aimed to characterize the chemical composition of pyroligneous acid (PA) obtained from slow pyrolysis of the clone GG100 of *Eucalyptus urophylla* × *Eucalyptus grandis*. The efficiency of extraction of organic compounds by using different solvents—dichloromethane (DCM), diethyl ether (DE) and ethyl acetate (EA)—was evaluated. Wood discs were collected and carbonized at a heating rate of 1.25 °C/min until 450 °C. Pyrolysis gases were trapped and condensed, yielding a crude liquid product (CLP), which was refined to obtain pure PA. Then liquid–liquid extraction was carried out. Each extracted fraction was analyzed by GC-MS and the chemical compounds were identified. Experimental results showed that a larger number of chemical compounds could be extracted by using DCM and EA in comparison to diethyl ether DE. A total number of 93 compounds were identified, with phenolic compounds being the major group, followed by aldehydes and ketones, furans, pyrans and esters. Higher contents of guaiacol, phenol, cresols and furfural seem to explain the antibacterial and antifungal activity shown by PA, as reported previously in the literature. Experimental data indicated that the organic phase extracted from GG100 PA consists of a mixture of compounds similar to liquid smokes regularly used in the food industry.

## 1. Introduction

The forest sector is important to Brazilian agribusiness, contributing to the production of goods and services and generation of employment opportunities. A chain of direct and indirect providers contributes to a flourishing forest sector that continuously requires raw materials, power, water and heavy machinery, banking and communication facilities, etc. Currently, Brazil has 7.8 million hectares of planted forests, of which nearly 5.6 million hectares correspond to eucalyptus forests, with 1.1 million hectares dedicated exclusively to the production of firewood for making charcoal [1]. In 2015, Brazilian consumption of charcoal reached 4.6 million metric tons. The metallurgy sector, including pig iron, steel, iron alloys and silicon industries, consumed about 80% of charcoal. This sector produces charcoal in industrial facilities, mostly working with partial-combustion masonry kilns that do not recover byproduct gases and condensable compounds. Only charcoal is considered important in this process. Currently, mass and energy yields can be very low in such conventional kilns, about 30 and 50%, respectively, when byproducts are not recovered [2,3]. This means that millions of tons of chemicals are lost to the atmosphere as gases without any recycling.

One of the most important liquid products from wood pyrolysis is pyroligneous acid (PA). According to Souza et al. (2012) [4], pyrolysis liquids are referred to in the literature by terms such as pyroligneous tar, pyrolysis oil, bio-oil, bio-crude oil, bio-fuel oil, liquid wood, wood oil, liquid smoke, pyroligneous acid and wood distillates. However, a corrective remark must be inserted here since the expressions pyrolysis liquids and wood distillates by themselves are too comprehensive to be used as synonyms of PA. In turn, pyroligneous tar, pyrolysis oil, bio-oil, bio-fuel and bio-crude oil are commonly used to refer to the heavier oily and combustible portion of liquid products from flash pyrolysis of lignocellulosic raw materials, as pointed out by several authors [5,6,7] among many others. From a similar standpoint, the term liquid smoke also cannot be used as a synonym for PA since the so-called commercial liquid smoke is a result of fractionation, purification or concentration of carbonization liquid products, giving aqueous, oily or dry powder products [8]. This means that the use of the expression liquid smoke to characterize PA is not correct, while there are several types of LQ with properties quite distinct from each other. So, in order to standardize terms and to avoid ambiguity, it is better to consider both pyroligneous acid (PA) and wood vinegar as being the aqueous portion obtained from carbonization of wood and other lignocellulosic raw materials, as defined by several authors [9,10,11,12,13].

Charcoal making usually produces, as byproducts, non-condensable gases, tar and pyroligneous acid (hereafter referred to as PA). PA yields and chemical composition can widely vary depending on the type of the woody raw material and the pyrolysis process parameters, e.g., final temperature and heating rate [11,14]. Usually, PA is recovered from charcoal kilns by trapping the carbonization gases through a condensing unit or even simple metal pipes. After a certain interval (which can vary from days to weeks), wood tar, which is a heavier black oily fraction, decants at the bottom of the container and separates from PA. Agricultural uses of PA date back to the 1930s in Japan, when the product started to be applied as an antifungal and antibacterial agent on crops [12]. A large number of papers and technical reports show the undeniable efficiency of PA for soil conditioning [14,15,16] and for pest control in pure form or combined with conventional pesticides [4,12,17,18]. Additionally, PA has been successfully used to control fungal and bacterial diseases in plants [14,19] and as a herbicide [16]. Finally, PA is used as a plant growth enhancer, significantly stimulating the development of roots, stems, leaves, flowers, tubers and fruits, and also improving the sweet taste of fruits [14,20].

Despite being well-known worldwide and extensively used in agriculture, in Brazil the technical applications of PA are restricted mainly to small organic farming operations, leaving a much larger agribusiness market that can obtain benefits from the product. A significant element explaining the resistance to using PA is the ambiguous way of thinking, because many people strongly believe that PA can cause cancer and other diseases. This idea is related to the fact that PA can sometimes contain traces of wood tar, which is confused with coal tar, an undeniably toxic and carcinogenic compound [21]. Thus, disambiguation must be accomplished to enlighten people regarding the safety of using *Eucalyptus* PA. Besides this, there is a lack in the literature regarding the following points. (i) Research and technical works do not specify in a clear way what carbonization parameters were used to obtain the PA fraction, such as final temperature and heating rate; (ii) Scientific articles only rarely correlate the antibacterial and fungicidal properties of PA with the chemical compounds present in it; (iii) A significant number of Brazilian eucalyptus forests were established with clones of a hybrid from *Eucalyptus urophylla × Eucalyptus grandis* (cited in the national literature as *Eucalyptus urograndis*). Among these clones, GG100 stands out as one of the most used to form energy forests and for charcoal making in Brazil. However, despite such widespread application, there is no information about the chemical composition of the PA obtained from it. The present work aims to fill the gaps mentioned above, by clearly identifying the conditions applied to obtain PA from GG100 wood as raw material, as well as the standard method used for PA purification. Another specific goal is to identify the main components of PA by gas chromatography combined with mass spectrometry (GC-MS) and to present a brief review of PA’s compounds, including their main technical uses and possible biological effects.

## 2. Results and Discussion

Gravimetric yields (GY) of charcoal, total condensed liquids (crude PA) and non-condensable gases (NCG) from carbonization runs are shown in Table 1. These results obtained from charring runs agree with experimental data reported by Oliveira et al. (2006) [22], Pereira et al. (2013) [23] and Santos et al. (2013) [24], who all obtained similar yields and charcoal with acceptable quality in terms of industrial charcoal making.

Table 2 shows the gravimetric yields of purified PA achieved by vacuum distillation of crude PA (total pyrolysis liquids) of *Eucalyptus urograndis* GG100 clone. The results attained by purification of crude PA were satisfactory for commercial purposes. Table 3 reports the properties of GG100 PA after distillation; they are in compliance with properties normally cited for PA in the literature cited here.

**Figure 1:**
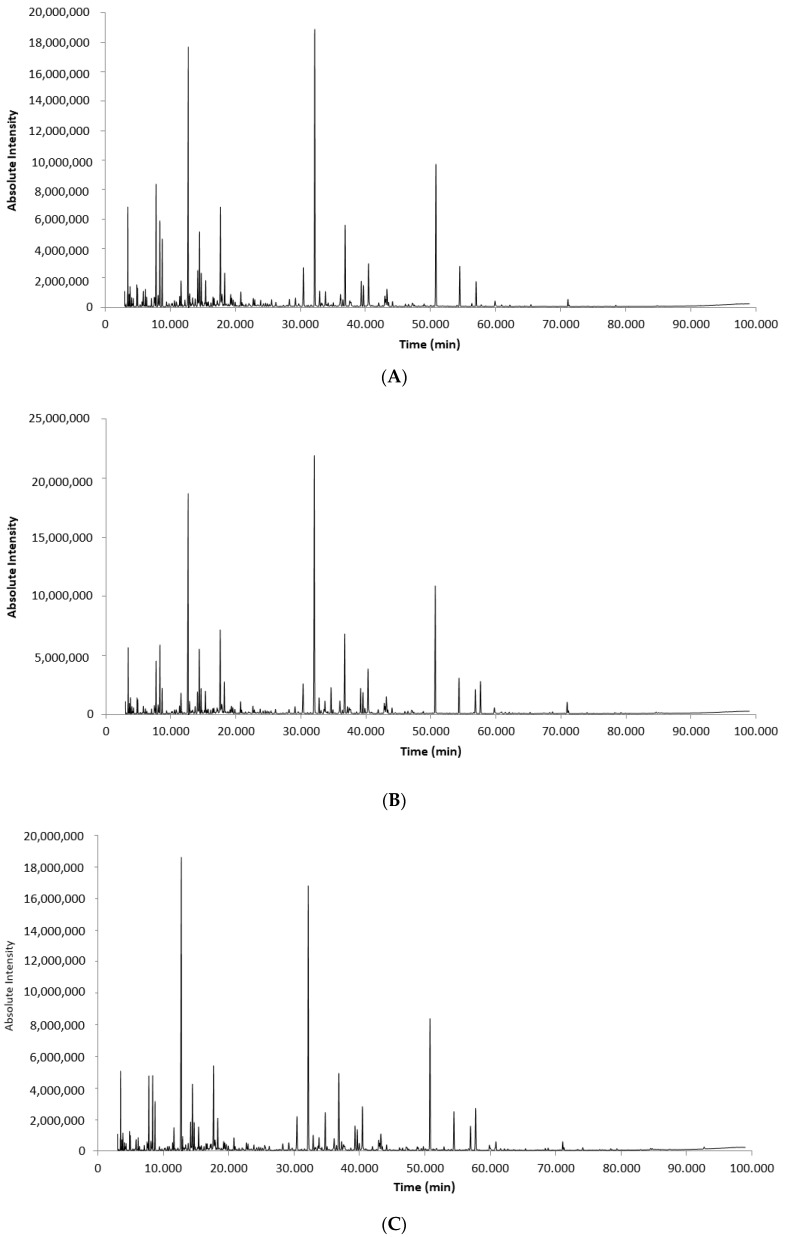
Total ion chromatograms of extracts obtained by using dichloromethane (**A**), diethyl ether (**B**) and ethyl acetate (**C**).

Figure 1 shows the total ion chromatograms of the *Eucalyptus urograndis* PA extracted with dichloromethane, diethyl ether and ethyl acetate (A, B and C, respectively). The profile of the chromatograms is similar, but the amount and type of compounds extracted by each solvent are different (as discussed shortly). Table 4 identifies the compounds extracted by each solvent. A total number of 93 main compounds were identified, divided into the following groups: phenolic compounds, aldehydes and ketones, furans and pyrans.

Regarding the sample extraction procedure, the addition of ammonium hydroxide to PA aqueous samples is an important step because it decreased the concentration of acetic acid extracted in the organic phase. It also increased the ionic strength of the solution, making the organic compounds less soluble in the aqueous phase and thus improving the efficiency of the liquid–liquid extraction, allowing detection of compounds even at low levels. As shown in Table 5, 65, 56 and 75 compounds were extracted by dichloromethane, diethyl ether and ethyl acetate, respectively, corresponding to 69.9%, 60.2% and 80.6% of the 93 compounds identified in GG100 clone PA. Therefore, in terms of number of extracted compounds, ethyl acetate was the most efficient solvent, followed by dichloromethane and diethyl ether. In terms of exclusive extraction, only four compounds were selectively extracted by diethyl ether (4.3% of the 93 compounds identified), against ten and 12 compounds exclusively extracted with dichloromethane and ethyl acetate. The results achieved are in accordance to those cited previously by Rungruang and Junyapoon (2010) [25], who found the same order of efficiency for these three solvents for the extraction of phenolic compounds from Eucalyptus wood. 

A significant number of the compounds identified here are the same cited in the literature as being components of liquid smoke used in the food industry [8,26,27,28]. Phenolic compounds are an important group identified both in PA and liquid smoke [8,26]. The same compounds are reported as being present in PA from different origins as well [4,13,29]. The high contents of guaiacol, phenol, cresols and furfural present in GG100 PA explain its antibacterial/antifungal activities, as pointed out by others authors [29], who stated that antibacterial and antifungal activity of PA from different sources cannot be attributed to a single compound, but instead to a combination of several ones.

A brief review of literature on some of the major components of GG100 PA is included from this point ahead, highlighting their properties, occurrence and biological effects, following the order of elution of the compounds shown in Table 4. 2-methyl-2-pentanol is flavoring agent used in food [30]. Cyclopentanone is a flavoring agent usually found in commercial liquid smoke [8,26]. 2-cyclopenten-1-one and its derivatives are food additives and are also found in commercial liquid smoke [8]. Furfural (2-furaldehyde) is a flavoring agent found in allspice and in commercial liquid smoke products [8,26]. 3-methyl-2-cyclopenten-1-one is a flavoring agent found in commercial liquid smoke [8,26]. 2-acetyl-furan is used in flavor compositions and contributes to aroma of many foods and beverages [30]. Tetrahydro-2-furanmethanol is a flavoring agent and food additive found in fermented soya hydrolysate (shoyu) and in liquid smoke [8,26,31]. 5-methyl-2-furancarboxaldehyde is a flavoring agent found in pepper, isolated from brown algae and other plant sources [31] and present in liquid smoke [8]. Methyl-2-furoate and 3-methyl-1,2-cyclopentanedione are flavoring agents [31]. Guaiacol is used medicinally as an expectorant, antiseptic, and local anesthetic [30]. Guaiacol and its derivatives have antioxidant properties and are present in commercial liquid smoke [8,26]. 4-methyl-2-methoxy-phenol (creosol) is a flavoring agent present in several foods and beverages [31] and in liquid smoke [8,26].

Phenol has some therapeutic value as a fungicide, antiseptic and disinfectant [31], with activity against a wide range of microorganisms including some viruses. Phenol is also one of the components of commercial liquid smoke products [8]. Cresols (*o*, *m* and *p*-cresol or 2,3 and 4-methy-phenol) are used as local antiseptics, parasiticides, disinfectants and as intestinal antiseptics [31], and are also present as components in liquid smoke products [8,26]. Other compounds present in PA, such as 4-ethyl-2-methoxy-phenol, maltol, 4-oxo-methyl-esther pentanoic acid (methyl levulinate), 2,6-dimethoxy-phenol (syringol) and its derivatives and xylenols, are used as flavoring agents [8,26,30].

Regarding the presence of mutagenic compounds, specifically polycyclic aromatic hydrocarbons (PAHs), several authors have pointed out that by removing wood tar from PAs, the PAHs are concomitantly removed. PAHs have a strong environmental impact because of their mutagenic and carcinogenic [32] character, which makes detection of these compounds important in processed foods, drinking water, air, etc. for health safety reasons. Liquid products from pyrolysis have been assessed to establish their acute toxicity and genotoxicity, but the results showed that crude pyrolysis liquids of eucalyptus wood had no mutagenic properties [33] because PAHs are strongly adsorbed to the pitch fraction and are not bioavailable. Furthermore, Pakdel and Roy (1988) [34] demonstrated that PAHs are thoroughly mixed with tar pitch, and only traces of them remained in the pure PA as oily tar fraction separated from aqueous PA. A feature that stands out in the chemical composition of PA is the presence of *N*-nitrosodimethylamine (11th compound in Table 4). This compound can be formed during the cooking of foods, especially cured meats and fish, which contain sodium nitrite as a preservative, but it is also found in several vegetables, cheeses, alcoholic beverages and fruits. It has been found to induce tumor formation in experimental animals, indicating it may also be a human carcinogen [35]. Likewise, phenol is reported as having suspected mutagenic properties [36]. The literature cited here also shows that for agriculture uses, PA is usually greatly diluted before applying the product, normally from 1:200 to 1:1.000 depending on the crop. Even though PA is used in diluted form, there is no dose level fixed for potentially carcinogenic substances that allows classifying them as not harmful. Thus, attention may be required regarding persistence times of *N*-nitrosodimethylamine and phenol in fruits and vegetables after PA is applied on crops.

Decantation is the most frequently used method for purification of PA and removing wood tar [11], but the method is a long-term strategy because it sometimes requires several months to achieve acceptable separation of PA from wood tar. Distillation is another method that can provide PAs free of PAHs in standardized quality with reliability and reproducible operational conditions [10]. There are still other methods currently used to eliminate PAHs from commercial liquid smoke flavorings [37] that most likely could be applied to refine PAs due to the close similarity in terms of chemical composition. On the other hand, phenolic compounds from *Eucalyptus* pyrolysis can be toxic to living organisms and cells, but they do not present any mutagenic character, as demonstrated by Pimenta et al. (2000) [33]. In general, phenolic compounds are widely used in clinical dentistry as sedatives, disinfectants and medication because despite being cytotoxic agents, they have no mutagenic effect [38].

Based on these observations about GG100 PA’s chemical composition, it is not evident that since the product likely is free of PAHs after purification, it can be applied without any concern in agriculture or as additive in animal feed, because of the presence of phenols and *N*-nitrosodimethylamine in its chemical composition. Further research should be carried out to isolate and specifically test the mutagenic character of crude GG100 PA and its phenolic fraction. Effective methods to isolate the phenolic fraction from PA are reported in the literature [29,39].

## 3. Materials and Methods

### 3.1. Production and Purification of PAs

The wood for the experiment was obtained from a planted forest tended by the Agricultural Sciences Unit of Federal University of Rio Grande do Norte, located in the city of Macaíba, Rio Grande do Norte. The procedures for log collection and wood sampling followed the method described by Santos et al. (2013) [24]. Wood samples consisted of 2-cm-thick disks, each divided into four wedges. Wood wedges were oven dried for 48 h at 103 ± 1 °C until 0% moisture content. Then they were placed in a metal container in batches of about 500 g and were carbonized using a laboratory furnace. The furnace was equipped with a device designed to trap and collect the condensable portion of pyrolysis gases, and during all carbonization runs, the condenser was water cooled and maintained at 25 °C. Ten charring runs were carried out at a heating rate of 1.25 °C/min until final temperature of 450 °C, which was held for 30 min. After the charring runs, the total condensed liquids were immediately stored in a refrigerator at 2 °C until use. The liquids from 10 pyrolysis runs were combined to form a composite sample. From the experimental data, the yields of charcoal, total condensed liquids and non-condensable gases (NCG) were determined. Yields of NGC were obtained by difference. Then the composite sample of condensed liquids was bi-distilled under a 1.0 mm HG vacuum at 100 °C to obtain the purified PAs, with the distillation process being interrupted as soon as the temperature reached 102–105 °C. Wood tar and heavy oils from each distillation were discarded. After PA purification, the following properties were determined: pH; titratable acidity by using a Metrhom Titrando device (Herisau, Switzerland) 0.1 mol L^−1^ NaOH titration solution; density (Koehler K86201 automatic density meter, Bohemia, NY, USA); and color.

### 3.2. GC-MS Analysis of PA

First, 1.5 mL of concentrated ammonium hydroxide solution (Ammonia Solution UN 2672, Caledon, ON, Canada) was added to 5 mL aliquots of the aqueous samples of PA to increase the pH to around 5. Then extractions were carried out by adding 3 mL of the different solvents separately: dichloromethane (Tedia, Aparecida de Goiânia, Brazil), diethyl ether and ethyl acetate (Merck, Kenilworth, NJ, USA). All the solvents were HPLC grade. Three extracts were produced, respectively. After liquid-liquid extraction, 1 mL of the organic fraction obtained by each solvent was transferred to a GC vial and was promptly analyzed.

The GC-MS analyses of the samples were carried out with a Shimadzu QP 2010 system. The separation was performed in a CP-Wax column (Restek 52 DB, 30 m length, 0.25 mm diameter, 0.25 μm film thickness), keeping the injector temperature at 250 °C. The chromatographic runs were carried out in order to achieve the best separation of compounds, until the following routine of analysis could be defined. The samples (1 µL) were injected in a split ratio of 1:10, and the oven temperature program was 50 °C for 2 min, 2 °C min^−1^ from 50 to 240 °C, maintained for 2 min. Helium was used as a carrier gas at a constant flow rate of 1 mL min^-1^. Major (>20% area) and minor compounds (~0.02%) were detected and identified based on their characteristic mass spectra by comparison with the NIST library. All of the chemical compounds reported here had mass spectrum similarity above 85%.

After chemical identification of the compounds present in each extract sample, we searched the literature to determine the main uses and their biological effects. The goal here was not to perform an exhaustive review, but only a brief appraisal to highlight possible effects of PA components on human and animal health.

## 4. Conclusions

This work describes the efficiency of the liquid–liquid extraction of PA from Eucalyptus GG100 clone with dichloromethane, diethyl ether and ethyl acetate, followed by analysis of the extracts by GC-MS to identify the main compounds. The GG100 clone is planted in large scale in Brazil and the main components of its PA have antibacterial, antifungal and antioxidant properties, of interest for agricultural uses. Higher contents of guaiacol, phenol, cresols and furfural seem to explain the antibacterial and antifungal activity shown by PA, as reported previously in the literature. Besides that, we found that GG100 PA consists of a mixture of chemicals very similar to those in liquid smoke additives regularly used in the food industry. However, the presence of *N*-nitrosodimethylamine and phenol in PA composition may require attention regarding persistence times of these compounds in plants and the environment in case of applying PA on edible crops, since those two compounds have been found to be carcinogenic and are suspected of being carcinogenic, respectively.

## Figures and Tables

**Table 1:** Gravimetric yields from carbonization of *Eucalyptus urograndis* GG100 clone.

Species	Gravimetric Yields (%)		
	Charcoal	Total Condensed Liquids	NCG
*Eucalyptus urograndis* GG100 clone	35.3	42.4	22.3

* NGC—non-condensable gases.

**Table 2:** Gravimetric yields of purified PA from distillation.

Species	Gravimetric Yields (%)	
	Based on TPL * Mass	Based on Initial Dry Wood Mass
*Eucalyptus urograndis* GG100 clone	70.1	27.4

* Total pyrolysis liquids.

**Table 3:** Properties of GG100 PA after distillation.

Property	
Color	Yellow
pH	2.85
Titratable acidity	0.03342 g NaOH/g sample
Density	1.032 g cm^−3^

**Table 4:** Chemical compounds identified in the PA from *Eucalyptus urograndis* GG100 for dichloromethane, diethyl ether and ethyl acetate.

					Extraction Solvent								
					Dichloromethane			Diethyl Ether			Ethyl Acetate		
Identified Peak ^#^	Compound	Molecular Formula	Average Mass (Da)	Similarity (%)	OC	RT (min)	Area (%)	OC	RT (min)	Area (%)	OC	RT (min)	Area (%)
1	2-methyl-2-pentanol	C_6_H_14_O	102.175	87		3.178	0.05	*	*	*	*	*	*
2	Cyclopentanone	C_5_H_8_O	84.116	98		3.483	2.39		3.475	1.83		3.467	1.64
3	2-methyl-cyclopentanone	C_6_H_10_O	98.143	90		3.671	0.33		3.616	0.32		3.654	0.25
4	Tetrahydro-2,2-dimethoxy-furan	C_6_H_12_O_3_	132.158	93		3.838	0.60		3.782	0.36		3.820	0.39
5	3-methyl-cyclopentanone	C_6_H_10_O	98.143	89		4.051	0.31		3.991	0.29		4.032	0.19
6	2-methyl-pyridine	C_6_H_7_N	93.127	96		4.166	0.07	*	*	*	*	*	*
7	2-(methoxymethyl)-furan	C_6_H_8_O_2_	112.127	97		4.863	0.58		4.794	0.50		4.843	0.48
8	Tetrahydro-2,5-dimethoxy-furan	C_6_H_12_O_3_	132.158	96		4.968	0.50		4.900	0.43		4.947	0.39
9	2-methyl-propanoic anhydride	C_8_H_14_O_3_	158.195	95		5.660	0.13	*	*	*	*	*	*
10	1,4-dioxen	C_4_H_6_O_2_	86.089	88		5.855	0.73	*	*	*	*	*	*
11	*N*-nitrosodimethylamine	C_2_H_6_N_2_O	74.082	94		6.192	0.60		6.085	0.19		6.161	0.41
12	5-methylhexahydro-4*H*-1,3-benzodioxin-4-one	C_9_H_16_O	140.223	85	*	*	*		7.032	0.23	*	*	*
13	2,4-hexadienal	C_6_H_8_O	96.127	87		7.120	0.32	*	*	*	*	*	*
14	3-pentanol	C_5_H_12_O	88.148	93	*	*	*		7.556	0.14	*	*	*
15	1-methoxy-2-butanol	C_5_H_12_O_2_	104.148	91	*	*	*	*	*	*		7.618	0.28
16	4-hydroxy-3-hexanone	C_6_H_12_O_2_	116.158	88		7.646	0.38	*	*	*	*	*	*
17	2-cyclopenten-1-one	C_5_H_6_O	82.101	97		7.821	4.59		7.713	2.33		7.778	2.70
18	3,5-dimethyl-cyclohexanol	C_8_H_16_O	128.212	85		8.137	0.56		8.040	0.34		8.134	0.38
19	2-methyl-2-cyclopenten-1-one	C_6_H_8_O	96.127	97		8.404	3.38		8.307	3.14		8.368	2.90
20	1-hydroxy-2-butanone	C_4_H_8_O_2_	88.105	98		8.766	2.62		8.650	1.10		8.725	1.85
21	2-hydroxy-methyl ester-butanoic acid	C_5_H_10_O_3_	118.131	92		9.431	0.17	*	*	*		9.399	0.16
22	2-cyclohexen-1-one	C_6_H_8_O	96.127	97		10.654	0.31	*	*	*	*	*	*
23	3-furaldehyde	C_5_H_4_O_2_	96.084	94		11.442	0.38		11.324	0.29		11.405	0.30
24	Butanoic acid, 2-ethylcyclohexyl ester	C_12_H_22_O_2_	198.302	85	*	*	*	*	*	*		11.617	1.08
25	3-methyl-butanoic acid	C_5_H_10_O_2_	102.132	90		12.247	0.25	*	*	*	*	*	*
26	Furfural	C_5_H_4_O_2_	96.084	99		12.764	11.24		12.655	13.92		12.747	15.67
27	3,4-dimethyl-2-cyclopenten-1-one	C_7_H_10_O	110.154	96		13.006	0.48	*	*	*		12.953	0.14
28	2,3,4-trimethyl-2-cyclopenten-1-one	C_8_H_12_O	124.180	91		13.841	0.25		13.725	0.30		13.796	0.26
29	3-methyl-2-cyclopenten-1-one	C_6_H_8_O	96.127	95		14.214	1.71		14.087	1.15		14.167	1.23
30	2-acetylfuran	C_6_H_6_O_2_	110.111	98		14.490	3.49		14.361	3.57		14.445	3.12
31	Tetrahydro-2-furanmethanol	C_5_H_10_O_2_	102.132	93		14.759	1.59		14.637	1.40		14.714	1.36
32	1-isopropyl-1-cyclopentene	C_8_H_14_	110.197	90	*	*	*	*	*	*		15.232	0.08
33	2,3-dimethyl-2-cyclopenten-1-one	C_7_H_10_O	110.154	95		15.435	1.24		15.310	1.15		15.389	1.08
34	3,4,5-trimethyl-2-cyclopenten-1-one	C_8_H_12_O	124.180	91		16.260	0.14	*	*	*		16.215	0.16
35	2-Butanone, 1-(acetyloxy)-	C_6_H_10_O_3_	130.142	93		16.741	0.33	*	*	*		16.693	0.27
36	1-acetylcyclohexene	C_8_H_12_O	14.183	85	*	*	*	*	*	*		17.187	0.15
37	3-methyl pyrrole	C_5_H_7_N	81.116	89	*	*	*	*	*	*		17.320	0.18
38	2,3-pentanedione	C_5_H_8_O_2_	110.116	88		16.570	0.42		*	*		16.528	0.30
39	3,4,4-trimethyl-2-cyclopenten-1-one	C_8_H_12_O	124.180	85		17.236	0.18	*	*	*	*	*	*
40	5-methyl-2-furancarboxaldehyde	C_6_H_6_O_2_	110.111	98		17.720	5.12		17.586	5.26		17.667	4.20
41	Pentanoic acid, 4-oxo-, methyl ester	C_6_H_10_O_3_	130.142	88		17.937	1.01	*	*	*	*	*	*
42	Methyl-2-furoate	C_6_H_6_O_3_	126.110	98		18.362	1.65		18.223	1.91		18.316	1.59
43	Butyrolactone	C_4_H_6_O_2_	86.089	95	*	*	*	*	*	*		19.218	0.39
44	4-hydroxy-butanoic acid	C_5_H_10_O_3_	118.131	96		19.289	0.57	*	*	*	*	*	*
45	3-ethyl-2-cyclopenten-1-one	C_7_H_10_O	110.154	87		19.449	0.50	*	*	*	*	*	*
46	2-acetyl-5-methylfuran	C_7_H_8_O_2_	124.137	92		19.665	0.24	*	*	*		19.617	0.30
47	Methylbenzoate	C_8_H_8_O_2_	136.148	90		19.871	0.18	*	*	*		19.926	0.23
48	2,5-dihydro-3,5-dimethyl-2-furanone	C_6_H_8_O_2_	112.127	96		20.845	0.71		20.704	0.72		20.796	0.63
49	Acetophenone	C_8_H_8_O	120.148	88		21.018	0.15	*	*	*		20.972	0.17
50	5-methyl-2(5*H*)-Furanone	C_5_H_6_O_2_	98.100	90	*	*	*	*	*	*		22.043	0.09
51	3-ethyl-2-hydroxy-2-ciclopenten-1-one	C_7_H_10_O_2_	126.153	92		22.757	0.38		22.615	0.46		22.707	0.39
52	2-furanmethanol (furfury alcohol)	C_5_H_6_O_2_	98.100	98		22.987	0.27		22.844	0.18		22.944	0.28
53	3-methyl-2(5*H*)-furanone	C_5_H_6_O_2_	98.100	92		23.984	0.24	*	*	*		23.839	0.28
54	4,5-dimethyl-4-hexen-3-one	C_8_H_14_O	126.196	88	*	*	*		24.505	0.21		24.595	0.15
55	2(5*H*)-furanone	C_4_H_4_O_2_	84.073	92		25.561	0.25	*	*	*		25.502	0.22
56	2-propylcyclohexanone	C_9_H_16_O	140.223	87	*	*	*	*	*	*		25.603	0.17
57	3-methyl-4-hexen-2-one	C_7_H_12_O	112.170	85	*	*	*	*	*	*		26.692	0.10
58	1,2-dimethoxy-benzene (veratrol)	C_8_H_10_O_2_	138.164	92		26.231	0.14		26.089	0.28		26.184	0.20
59	Methyl 4-hydroxybutanoate	C_5_H_10_O_3_	118.131	91		28.308	0.32	*	*	*		28.262	0.36
60	2,4-Dimethyl-1,3-cyclopentanedione	C_7_H_10_O_2_	126.153	89		29.232	0.44		29.089	0.51		29.180	0.51
61	3-methyl-1,2-cyclopentanedione	C_6_H_8_O_2_	112.127	97		30.487	2.37		30.331	2.42		30.430	2.25
62	2-methoxy-phenol (guaiacol)	C_7_H_8_O_2_	124.137	98		32.214	16.49		32.016	19.64		32.161	16.31
63	3-methyl-2-methoxy-phenol	C_8_H_10_O_2_	138.164	96		32.950	0.82		32.795	1.15		32.898	0.92
64	Furan-2-carbaldehyde, (*N*’-nitroamidino)hydrazone	C_6_H_7_N_5_O_3_	197.054	85	*	*	*	*	*	*		34.748	2.29
65	2,6-dimethyl-phenol	C_8_H_10_O	122.164	97	*	*	*		34.921	0.21		35.027	0.19
66	2-methoxy-5-methyl-phenol	C_8_H_10_O_2_	138.164	97		36.158	0.63		35.998	0.80		36.107	0,63
67	Maltol	C_6_H_6_O_3_	126.110	98		36.528	0.33		36.367	0.39		36.477	0.23
68	4-methyl-2-methoxy-phenol (creosol)	C_8_H_10_O_2_	138.164	97		36.880	4.73		36.722	5.91		36.830	4.87
69	Phenol	C_6_H_6_O	94.111	98		39.350	1.41		39.185	1.72		39.301	1.43
70	2-methyl-phenol (*o*-cresol)	C_7_H_8_O	108.138	97		39.700	1.16		39.541	1.49		39.651	1.21
71	4-ethyl-2-methoxy-phenol	C_9_H_12_O_2_	152.190	98		40.487	2.38		40.327	3.11		40.434	2.61
72	4-methyl-phenol (*p*-cresol)	C_7_H_8_O	108.138	97		42.984	0.54		42.818	0.77		42.931	0.67
73	2,6-dimethyl-phenol (2,6-xylenol)	C_8_H_10_O	122.164	95		43.120	0.34		42.959	0.32		43.072	0.31
74	3-methyl-phenol (*m*-cresol)	C_7_H_8_O	108.138	96		43.302	1.23		43.135	0.88		43.250	0.49
75	2,5-dimethy-phenol (2,5-xylenol)	C_8_H_10_O	122.164	87	*	*	*	*	*	*		43.267	0.58
76	3,4-dimethoxy-phenol	C_8_H_10_O_3_	154.163	91		43.531	0.21		43.369	0.21		43.480	0.17
77	4-propyl-2-methoxy-phenol	C_10_H_14_O_2_	166.217	92		44.172	0.24		44.013	0.45		44.121	0.37
78	2,4-dimethyl-phenol (2,4-xylenol)	C_8_H_10_O	122.164	85	*	*	*		45.982	0.16		46.098	0.14
79	3-allyl-6-methoxy-phenol	C_10_H_12_O_2_	164.201	97	*	*	*		46.458	0.20		46.574	0.15
80	3,4-dimethyl-phenol (3,4-xylenol)	C_8_H_10_O	122.164	93	*	*	*		47.043	0.34	*	*	*
81	3-ethyl-phenol	C_8_H_10_O	122.164	94	*	*	*		47.350	0.09	*	*	*
82	3,5-dimethyl-phenol (3,5-xylenol)	C_8_H_10_O	122.164	87	*	*	*		48.834	0.19	*	*	*
83	4,5-dimethyl-imidazol	C_5_H_8_N_2_	96.130	98	*	*	*	*	*	*		49.749	0.21
84	2,6-dimethoxy-phenol (syringol)	C_8_H_10_O_3_	154.163	97		50.829	8.78		50.665	9.90		50.770	8.48
85	4-methyl-2,6-dimethoxy-phenol	C_9_H_12_O_3_	168.190	80	*	*	*	*	*	*		54.427	2.52
86	1,2,3-trimethoxy-benzene	C_9_H_12_O_3_	168.190	85		54.484	2.42		54.320	2.83	*	*	*
87	1,2,3-trimethoxy-5-methyl-benzene	C_10_H_13_O_3_	182.216	85		57.006	1.57		56.847	1.86		56.953	1.64
88	2,6-dimethoxy-4-allyl-phenol	C_11_H_14_O_3_	194.227	88	*	*	*		62.035	0.13		62.141	0.11
89	Guaiacyl acetone	C_10_H_12_O_3_	180.201	90	*	*	*		65.253	0.11		65.365	0.10
90	2-methyl-5-amino-benzoxazole	C_14_H_11_NO	209.243	88	*	*	*		68.719	0.13		68.834	0.12
91	2-acetyl-7-hydroxybenzofuran	C_10_H_8_O_3_	176.168	85	*	*	*	*	*	*		71.252	0.16
92	2-amino-1-naphthol	C_10_H_9_NO	159.185	89	*	*	*	*	*	*		74.131	0.17
93	Hydroquinone	C_6_H_6_O_2_	110.112	87	*	*	*		79.224	0.09		79.353	0.10

OC—Compound Occurrence in the respective extract: (^#^ positive occurrence—marked in blue; negative occurrence—marked with an asterisk).

**Table 5:** Efficiency of solvents in the extraction of organic compounds from GG100 PA.

Parameters	Extraction Solvent		
	Dichloromethane	Diethyl Ether	Ethyl Acetate
Total number of compounds extracted by the solvent	65	56	75
% of the total extracted compounds	69.9	60.2	80.6
Number of compounds exclusively extracted by the solvent	10	4	12
